# Non-specific chronic low back pain and physical activity: A comparison of postural control and hip muscle isometric strength: A cross-sectional study

**DOI:** 10.1097/MD.0000000000018544

**Published:** 2020-01-31

**Authors:** Muhsen B. Alsufiany, Everett B. Lohman, Noha S. Daher, Gina R. Gang, Amjad I. Shallan, Hatem M. Jaber

**Affiliations:** a Department of Physical Therapy; b Department of Allied Health Studies, School of Allied Health Professions, Loma Linda University, Loma Linda, CA; c Department of Physical Therapy, Faculty of Applied Medical Sciences, Taif University, Kingdom of Saudi Arabia; d Department of Physical Therapy, College of Rehabilitative Sciences, University of St. Augustine for Health Sciences, Austin, TX.

**Keywords:** hip strength, non-specific chronic low back pain, physical activity, postural control

## Abstract

Most research on sedentary lifestyle has focused on pain and disability, while neuromuscular outcomes (postural control and strength) have received less attention. The objective of the study was to determine whether low level of physical activity is negatively associated with measures of lower body muscular strength and postural control in individuals with and without non-specific chronic low back pain (NSCLBP).

Twenty-four subjects with NSCLBP (28.8 ± 5.9 years) and 24 age, gender, and body mass index matched healthy controls participated in the study. Subjects were sub-classified into 4 subgroups based on their physical activity level: Non-active NSCLBP; Active NSCLBP; Non-active healthy control; and Active healthy control. Each subgroup consisted of 12 subjects. Peak force of hip muscles strength was assessed using a handheld dynamometer. Postural control was assessed using computerized posturography and the Y Balance Test.

There was no significant group by physical activity interaction for strength and static and dynamic postural control, except for static control during left single leg stance with eyes closed (*P* = .029). However, there was a significant difference in strength and postural control by physical activity (*P* < .05). Postural control and peak force of hip muscles strength were significantly associated with physical activity (*r* ranged from 0.50 to 0.66, *P* < .001 and *r* ranged from 0.40 to 0.59, *P* < .05, respectively).

Postural control and hip strength were independently related to physical activity behavior. A sedentary behavior may be an important risk factor for impaired postural control and hip muscles strength, and that physical fitness is vital to neuromuscular outcomes.

## 1 Introduction

Low back pain (LBP) is a major health issue that causes more disability and global burden than any other conditions.^[1]^ It is one of the most common musculoskeletal disorders and it is estimated that approximately 60% to 80% of adults will experience LBP at some point in their lives. Ten percent of these cases will develop chronic low back pain (CLBP).^[2]^ CLBP is associated with increased medical expenditure, work absence, and loss of quality of life.^[3,4]^ In fact, the direct costs of medical expenditures and loss of work productivity related to back pain have been estimated to be as high as $635 billion annually in the United States alone.^[5]^ Nonetheless, 85% of CLBP disorders are categorized as non-specific chronic low back pain (NSCLBP) due to unknown source.^[6]^ Despite the recent attempts towards understanding the underlying mechanism; NSCLBP remains a disabling condition restricting daily physical activities and quality of life of the affected individuals.^[7]^

It has been suggested that a sedentary lifestyle, defined as prolonged sitting during work and leisure time with energy expenditures of below 600 MET min/week, is one of the risk factors for developing NSCLBP.^[8–13]^ Evidence has shown an inverse association between physical activity behavior and pain and disability in individuals with CLBP.^[14–16]^ In a prognostic study by Pinto et al,^[14]^ patients with CLBP who had a moderate or higher activity level at baseline showed less pain and disability at 12 months’ follow-up than those who were sedentary. In addition, NSCLBP patients who presented with higher levels of disability were found to have lower levels of physical activity.^[15,16]^ The increased physical disability was shown to impact postural control performance in sedentary women with NSCLBP.^[17]^

While the effect of low levels of physical activity on pain and disability is becoming clear, the possible effect on postural control outcomes has received less attention to date. A sedentary behavior may inadvertently cause reduced neuromuscular efficiency,^[18]^ increased skeletal muscles atrophy, and diminished muscle strength.^[19]^ This reduction of physical activity and the associated muscle weakening of the lower limbs might have significant negative consequences on postural control and functional performance,^[17,20–23]^ and could contribute to back pain. In fact, poor neuromuscular control has been identified as an important risk factor in the development of NSCLBP.^[24]^ For instance, individuals with NSCLBP have been shown to demonstrate an altered motor control of deep trunk muscles, leading to alteration and/or reduction of postural control strategies.^[25,26]^ These observed postural control behaviors have been suggested as one of the possible factors contributing to the disorder.^[27]^ In addition, previous research has reported that symptom free individuals, who presented with postural control strategies similar to that of LBP patients, were at a greater risk to develop NSCLBP.^[28]^

Maintenance of static and dynamic postural control is crucial for functional activities.^[29]^ In NSCLBP individuals, postural control might be deteriorated, and thus may affect the ability to perform daily activities safely and effectively. Although previous research has reported no difference in physical activity level between those with NSCLBP and healthy individuals,^[30]^ the type and quality of physical activity; however, were shown to be different and could influence disability.^[31,32]^ While most studies have investigated the association between physical activity and back pain/disability,^[14–16]^ little information is available regarding the influence of physical activity on the performance of motor tasks; specifically, postural control in individuals with NSCLBP. Important information could be gathered from direct measurement of postural control and physical activity level in this population. Such data may help to guide clinical practice in regards to fitness training interventions in this population.

The association between neuromuscular outcomes (postural control and strength) and physical activity in individuals with NSCLBP has to our knowledge not yet been established. The aim of this study, therefore, was to examine differences in postural control and strength among subgroups of physically active and inactive NSCLBP individuals and healthy controls, and to further investigate the association between postural control impairments and physical activity as measured by the International Physical Activity Questionnaire Short Form (IPAQ-SF), and between strength and physical activity. We hypothesized that postural control and hip strength is diminished in NSCLP individuals as compared to healthy controls, diminished in physically inactive as compared to physically active individuals, and that impaired postural control and strength will be associated with physical inactivity in NSCLBP.

## 2 Materials and methods

### 2.1 Study design and Setting

This was a cross-sectional study. Subjects were recruited by flyers, email, and word of mouth from a college campus and a community in Sothern California. All experimental procedures were conducted in the orthopedic laboratory at Loma Linda University, Department of Physical Therapy from September 2017 to November 2018.

### 2.2 Participants

Twenty-four subjects with NSCLBP and 24 age, Body Mass Index (BMI), and gender-matched healthy controls participated in this study. Each group consisted of 12 males and 12 females (age range, 20−45 years). All subjects read and signed a written informed consent approved by the Institutional Review Board of Loma Linda University before participation. Subjects with NSCLBP were included if they presented with LBP of at least 3/10 on the Numeric Pain Rating Scale (NPRS) for a duration of >3 months. Subjects in the control group had to be free of LBP for at least 1-year before participation and never had an episode of LBP that lasted more than 3 months in the past. Subjects in both groups were excluded if they had one of the following:

(1) pregnancy, including 6 months postpartum;
(2) a history of back or lower extremity surgery;
(3) radiating pain below the gluteal fold;
(4) trauma to the back or lower extremities for at least 3 months before the study;
(5) current lower extremity pain;
(6) neurological or vestibular disorders;
(7) consumed over the counter pain medication, drugs or alcohol within 24 hours before the study;
(8) physical activity score between 550 and 649 on the metabolic equivalent task questionnaire; or
(9) a BMI greater than 30 kg/m^2^_._

### 2.3 Assessment protocol

*The Numeric Pain Rating Scale (NPRS)* was used to measure pain intensity in the lower back region. It is a linear measurement on a straight 100 mm line with 10 mm intervals. The score ranges from 0 to 10, where “0” indicates no pain and “10” indicates the worst and most frequent pain imaginable. Subjects were asked to choose a number that best represents the intensity of their pain, with higher NPRS indicating higher severity of LBP. The NPRS has high validity (r ranging 0.64–0.84) and moderate reliability (r ranged from 0.60 to 0.77) in assessing pain.^[33]^

*The International Physical Activity Questionnaire - Short Form* was used as a self-reported measure to assess the level of physical activity. The IPAQ-SF is a 9-item scale that provides information on the amount of time (minutes) spent walking, in moderate and vigorous intensity activity, and sitting during the past 7 days. Frequency is measured by number of days per week and duration is measured in minutes per day for each activity. For scoring, the amount of metabolic equivalents task (METs)-minutes/week for each category was calculated by multiplying the number of minutes by 3.3 (walking), 4 (moderate), 8 (vigorous), or 1.3 (sitting). In addition, a total score was calculated by counting the METs-minutes of the first 3 categories together [Total physical activity MET- minute/week = (Walk METs × min × days) + (Moderate METs × min × days) + (Vigorous METs × min × days)]. Subjects whose scores are lower than 600 MET are classified as inactive, and those with scores equal or higher than 600 MET are classified as active. The IPAQ-SF has demonstrated good test-retest reliability (Intraclass correlation coefficient [ICC] = 0.80) and a moderate concurrent validity with the long form (Spearman's *ρ* = 0.67; 95% confidence interval [CI] [0.64–0.70]).^[34]^

After all subjects completed the IPAQ-SF questionnaire, they were categorized into 4 subgroups based on their levels of physical activity. In this study, an arbitrary cut off score of **>**649 MET was considered as physically active, and a score of **<**550 MET was considered as physically non-active. Subjects who scored between 550 and 649 were excluded from the study to control for any potential effects on the results.^[35]^ Groups were sub-classified as follows, Group A: Non-active, NSCLBP; Group B: Active NSCLBP; Group C: Non-active healthy control; and Group D: Active healthy control. Each subgroup consisted of 12 subjects, 6 males and 6 females. Following the sub-classification, all subjects underwent the following testing protocols:

### 2.4 Strength testing

Peak isometric hip flexors, extensors, abductors, and external rotators’ strength were measured bilaterally with a handheld dynamometer (MicroFet3, Draper, UT) using previously reported reliable muscle testing protocols.^[36–38]^ Prior to the testing trials, subjects performed 1 sub-maximal contraction practice trial to ensure adequate performance and stabilization. Three 5-seconds (maximum voluntary isometric contraction [MVIC]) measurement trials were completed for each muscle group with a 30-seconds rest period between each trial. Verbal encouragement was provided during each trial to ensure maximal effort. The same tester performed all measurements to ensure consistency, and muscle testing order was randomized to minimize bias. The peak force values were recorded in Newtons and expressed as a percentage of each subject's body mass.^[39]^ Normalized value data from the three trials were averaged and used for data analysis.

### 2.5 Dynamic balance testing

Dynamic balance was assessed using the Y-Balance Test (YBT) (FunctionalMovement.com, Danville) under the supervision of a certified practitioner. Following muscle strength testing, subjects received a 5-minute rest period. Subjects then viewed an instructional video on proper YBT performance. Subjects were then instructed to stand barefoot with the test foot on the stance plate with the toes of the test foot just behind the red start line while the non-test foot touched down lightly on the floor posterolaterally to the stance plate. Next, subjects were instructed to push the red target on the side of the reach indicator as far as possible in the desired direction and then, under control, return to the starting position. The testing order was as follows starting with the right limb;

(1) anterior (A) reach,
(2) posteromedial (PM) reach, and
(3) posterolateral (PL) reach.

The same sequence was then performed on the left limb. Four practice trials were allowed in each reach direction to familiarize subjects with the testing maneuvers to help stabilize their performance and maximize reach distance. Next, subjects performed three testing trials on each leg. An additional trial was given if necessary. Thirty seconds of rest were given between each reach trial and 60 seconds between each direction to minimize fatigue. A trial was discarded and repeated if the subject:

(1) touched the floor with the foot during the reach or the return phase,
(2) did not keep their hands on their waist,
(3) placed his/her foot or toes on top of the reach indicator to maintain balance during the reach (push) phase,
(4) unintentionally kicked the reach indicator to create momentum to advance the box, or
(5) failed to return to the starting position under control.

Measurements from the 3 testing trials in each direction were averaged and normalized to the subject's leg length [average reach distance (cm)/leg length (cm) × 100], which was measured manually from the most prominent aspect of the anterior superior iliac spine to the distal tip of the ipsilateral medial malleolus.^[35]^ The average reach distance for each direction was expressed as a percentage of leg length and used for analysis. A composite score was also calculated by dividing the sum of maximum reaches in each of the 3 directions by 3 times the leg length then multiplied by 100.

### 2.6 Static balance testing

Following dynamic balance testing, subjects were given another 5-minutes rest period. They were then asked to stand on one leg on the Balance Master (BM) force platform (NeuroCom International, Inc. Clackamas, OR) under each of the following four conditions: with eyes open and closed, for the right and left legs. Each condition was repeated three times for ten seconds each. A trial was discarded and repeated if the subject lost single leg stance balance or did not keep their hands on their waist. A maximum of three repetitions were allowed for each trial, and if the subject was unable to perform the task, a trial was recorded as a fail. Sway velocity (degrees/seconds) was recorded during each testing condition. Data collected from the three testing times under each condition was averaged and used for analysis.

### 2.7 Statistical analyses

Data was analyzed using SPSS version 24.0 (IBM Corp, Armonk, NY). A sample size of 60 participants (n = 15 per subgroup) was estimated using a medium effect size (Partial *η*2 = 0.1 or Cohen d = 0.8), level of significance (α = 0.05), and a power of 0.8. We were able to recruit 48 subjects (n = 12 per subgroup). Mean ± standard deviation (SD) was computed for continuous variables and frequencies (%) for categorical variables. Normality of the quantitative variables was assessed using Shapiro-Wilk test and boxplots. Mean age (years), BMI (kg/m^2^), bilateral isometric hip strength was compared between the 2 groups using independent *t* test. Distribution of gender by group type was examined using Pearson Chi Square test.

A 2 × 2 factorial analysis of variance (ANOVA) was used to compare the mean hip strength, reach distance, and sway velocity (degrees/sec) with eyes open and eyes closed by group type and physical activity. The primary analysis included a comparison between groups using the group x physical activity interaction effect. If the interaction was statistically significant, the difference was compared between groups at each physical activity level using independent *t* test. If the results of the interaction were not statistically significant, the between-groups comparisons were considered not statistically significant. However, Bonferroni post hoc test was conducted on the combined groups only if the main effect of physical activity was significant in the factorial ANOVA.

The secondary analysis included testing differences in mean reach distance and mean sway velocity (degrees/second) with eyes open and eyes closed for each group separately (within group) using independent *t* test. Pearson correlation coefficients were used to assess the linear relationship between postural control and physical activity, and between strength and physical activity. The level of significance was set at *P* ≤ .05.

## 3 Results

Fifty-five subjects were screened for eligibility, seven were excluded for not meeting the inclusion criteria (5 reported radiating pain below the gluteal fold and 2 had a recent lower extremity injury). Thus, 48 participants with a mean age 28.5 ± 5.0 years old and BMI of 25.3 ± 2.5 kg/m^2^ completed this study. Fifty percent of the participants were females (n = 24) and 50% were active (n = 24). The distribution of all quantitative variables was approximately normal. There was no significant difference in demographic and general characteristics between the 2 groups (Table 1). The NSCLBP group had a median (minimum, maximum) pain level on the day of testing of 3 (3,7).

**Table 1:**
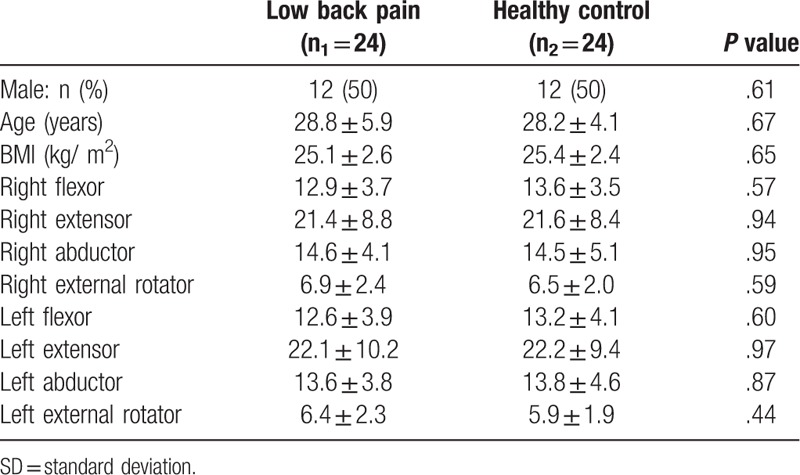
Mean ± SD of general characteristics by study group (N = 48).

### 3.1 Between group analysis

#### 3.1.1 YBT reach distance

Results of the 2 × 2 factorial ANOVA are displayed in Table 2. There was no significant group × physical activity interaction effect for all directions (*P* > .05). However, there was a significant difference in mean reach distance by physical activity level (active vs inactive) in all directions (right A, 68.5 ± 6.5 vs 62.9 ± 5.9, *P* = .004, partial *η*^2^ = 0.18; right PM, 112.1 ± 11.3 vs 98.1 ± 12.3, *P* < .001, *η*^2^ = 0.28; right PL, 110.1 ± 11.8 vs 94.1 ± 12.1 *P* < .001, *η*^2^ = 0.33; and right composite score, 99.2 ± 8.9 vs 86.9 ± 8.8, *P* < .001, *η*^2^ = 0.36, respectively). There were no interlimb differences (*P* > .05).

**Table 2:**
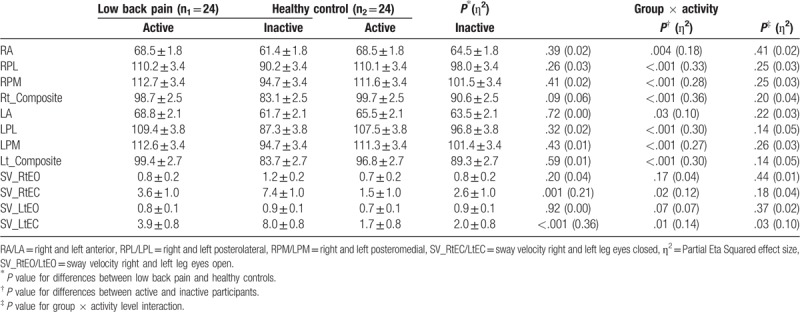
Mean ± standard error of balance scores among study group and activity level.

#### 3.1.2 BM sway velocity

Results of the 2 × 2 factorial ANOVA are displayed in Table 2. There was no significant group × physical activity interaction effect for sway velocity during right and left eye open and closed, and during left eye open (*P* > .05). However, there was a significant difference in mean sway velocity by physical activity level (active vs inactive) during both eyes closed conditions (right eye closed 2.6 ± 2.7 vs 5.0 ± 4.8, *P* = .02, *η*^2^ = 0.12; left eye closed 2.8 ± 2.8 vs 5.0 ± 4.3, *P* = .01, *η*^2^ = 0.14, respectively). There were no interlimb differences (*P* > .05). Nevertheless, there was a group × physical activity interaction effect in mean sway velocity during left eye closed (*P* = .03). Results of the independent *t* test showed that the difference was significant between inactive LBP individuals and inactive healthy controls (8.0 ± 4.3 vs 2.0 ± 0.8, *P* < .001, Cohen d = 1.9); however, no significant difference was found between physically active LBP individuals and active healthy controls (3.9 ± 3.7 vs 1.7 ± 0.3, *P* = .06).

#### 3.1.3 Hip muscle strength

Results of the 2 × 2 factorial ANOVA are displayed in Table 4. There was no significant group × physical activity interaction effect for strength in all muscles (*P* > .05). However, there was a significant difference in mean strength by physical activity level (active vs inactive) for all muscles except for right and left external rotator and left abductor (right flexor, 14.3 ± 3.7 vs 12.2 ± 3.1, *P* = .04, *η*^2^ = 0.09; right extensor, 24.2 ± 9.3 vs 13.8 ± 6.8, *P* = .03, *η*^2^ = 0.10; and right abductor, 16.1 ± 4.7 vs 13.0 ± 4.0 *P* = .02, *η*^2^ = 0.12, respectively). There were no interlimb differences (*P* > .05).

### 3.2 Within group analysis

Though significant interaction between group and physical activity was noted only in mean sway velocity during left eye closed, we sought to report within-group results for all postural control outcomes due to the clinical implications of the observed findings.

#### 3.2.1 YBT reach distance

Differences in reach distance within each study group are presented in Table 3. Among subjects with LBP, reach distance in all directions was significantly higher in physically active subjects compared to inactive subjects (*P* < .05, Cohen d ranged from 1.03 to 1.82). For healthy controls, however, only in the right PL direction, reach distance was significantly higher in physically active subjects compared to inactive subjects (*P* = .02, Cohen d = 1.04). For the other reach directions, there was no significant difference in mean reach distance by physical activity in healthy controls (*P* > .05).

**Table 3:**
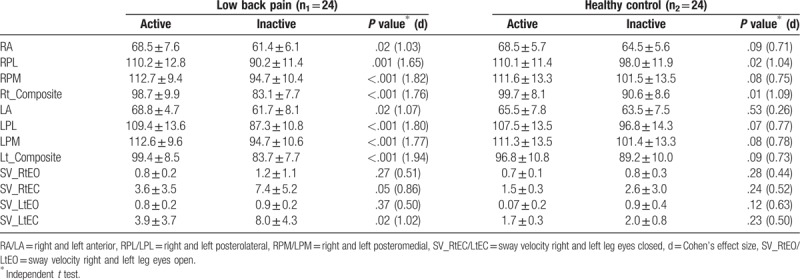
Mean ± SD of balance scores by activity level within each study group (N = 48).

**Table 4:**
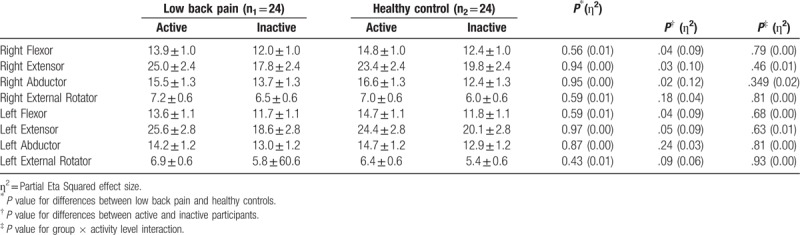
Mean ± standard error of strength (N) scores among study group and activity level (N = 48).

#### 3.2.2 BM sway velocity

Differences in mean sway velocity within each study group are presented in Table 3. Among subjects with LBP, sway velocity during right and left eyes closed was significantly better in physically active subjects compared to inactive subjects (*P* < .05, Cohen d = 0.86 and 1.02, respectively). For healthy controls, however, there was no significant difference in mean sway velocity during all testing conditions (*P* > .05).

### 3.3 Correlation

*For postural control,* among all participants, physical activity level positively correlated with reach distance in PM and PL directions of the YBT and for the composite score as well (*r* = 0.48, *P* = .001, *r* = 0.51, *P* < .001, and *r* = 0.53, *P* < .001, respectively). However, physical activity did not correlate with static control sway velocity (*P* > .05). Similarly, when looking at each group separately, there was a positive correlation between physical activity level and reach distance in PM and PL directions and composite score of the YBT for LBP group (*r* = 0.55, *P* = .005, *r* = 0.57, *P* = .004, and *r* = 0.61, *P* = .002, respectively) and for healthy controls (*r* = 0.50, *P* = .01, *r* = 0.66, *P* < .001, and *r* = 0.59, *P* = .002, respectively). In addition, there was a significant relationship between physical activity level and sway velocity during left eye closed in the LBP group; the higher the physical activity level, the lower (better) the sway velocity (*r* = −0.49, *P* = .02).

*For strength*, among all participants, hip flexor, extensor, and external rotator strength positively correlated with physical activity level (*r* = 0.31, *P* = .04, *r* = 0.51, *P* < .001, and *r* = 0.35, *P* = .02, respectively). However, when looking at each group separately, there was a positive correlation between physical activity level and hip flexor, extensor, abductor, and external rotator strength for healthy controls (*r* = 0.41, *P* = .05, *r* = 0.59, *P* = .003, *r* = 0.40, *P* = .05, and *r* = 0.44, *P* = .03, respectively). There was also a positive correlation between physical activity level and hip extensor and external rotator strength in the LBP group (*r* = 0.50, *P* = .03, and *r* = 0.50, *P* = .03, respectively).

## 4 Discussion

The present study aimed to compare differences in static and dynamic postural control and hip strength among subgroups of physically active and inactive NSCLBP individuals and healthy controls, and to further determine whether low level of physical activity is negatively associated with measures of lower body muscular strength and postural control. Our results revealed no significant group by physical activity interaction for hip muscles strength and postural control, except for static control during left single leg stance with eyes closed. However, we found significant differences in reach distances (for all Y-balance directions), sway velocity (during eye closed conditions), and all hip muscles strength (except external rotators and left abductor) by physical activity. Furthermore, we found a direct relationship between physical activity level and neuromuscular outcomes (postural control and strength).

### 4.1 Postural control

Postural control decreases in both LBP individuals and healthy controls in single leg stance with eyes closed conditions compared to eyes open, but only in eye closed conditions, a significant difference between LBP individuals and healthy controls is more distinct. In particular, inactive LBP individuals had significantly diminished static control as compared to inactive healthy controls. Nonetheless, the results indicated that static and dynamic postural control outcomes differed by physical activity level, with significantly lower scores in physically inactive individuals as compared to physically active peers. This was more evident in the LBP group during all YBT reach directions and during single leg static balance with eyes closed conditions. But for healthy controls, this was significantly evident only in the PL direction, despite the lower scores in the other directions. The PL direction is an extremely challenging direction,^[35]^ and being physically inactive could make it harder for a person to maintain balance in this direction even if he/she is otherwise healthy. The present study, however, did not find any interlimb differences or gender differences, which is in alignment with previous studies.^[40,41]^

Notwithstanding, a composite score of less than 89% has been reported to represent a reduced dynamic postural control and may place individuals at a risk for future injury.^[42,43]^ In this study, when comparing the LBP group to healthy controls, both groups had a mean score of greater than 89% on the right and left sides. However, when we studied the proportion of subjects who scored below 89% on the dominant side, within each group, the control group had only 3 subjects (12.5%) that scored below the 89% cut-off, while the LBP group had 10 subjects (44.7%) who scored below 89%, which is more than three times the number of subjects in the control group. More specifically, when examined at the subgroup level, the proportion of subjects that scored below 89% did not differ between active LBP and active controls [only 1 subject (8.3%) versus none (0%), respectively]. However, the proportion did differ considerably between inactive LBP and inactive controls [9 subjects (75%) vs 3 subjects (25%), respectively]. Similar findings were observed for the non-dominant left side.

Comparing our results with other research findings, however, is difficult as this was the first study to compare differences in postural control and hip strength among subgroups of physically active and inactive NSCLBP individuals and healthy controls. However, the clinical merits of our findings could be established by comparing the outcomes of the study with previous relevant studies. Impairment in static and dynamic postural control has been previously identified in individuals with NSCLBP.^[44–47]^ Our results however, showed that postural control was not significantly different between active individuals with and without NSCLBP. A possible explanation of this unexpected finding is that, despite the presence of LBP, active individuals sustained the same functional level of activity that is required to maintain postural stability as individuals without LBP, and thus might have masked the effects of pain on postural control. Similar to our findings, active individuals with LBP were found to have a postural control that is similar to those without LBP.^[40,48]^ It is possible, however, that impaired postural control is present in just a subgroup rather than in all LBP patients, meaning that some patients should not be expected to experience any change in postural control.

In contrast, however, postural control significantly differed between the inactive subgroups. To maintain stability, the body relies on integrated feedback from three sensory systems: visual, vestibular and somatosensory or proprioceptive.^[49]^ Individuals with LBP have been shown to demonstrate reduced proprioceptive feedback from mechanoreceptors of the trunk and hip joint, as a result of altered sensory input at the site of pain, which was suggested to affect postural control mechanism.^[50]^ Consequently, they usually adopt alternative postural control strategies and rely more on the visual and vestibular sources in order to cope with the new demands introduced by pain.^[51]^ Therefore, a reduction in visual feedback such that occurs during the eyes closed static balance conditions and the posterior directions of the YBT would further limit their postural control strategies. Visual cues are required to orient the body in space and to provide feedback for the reaching leg.^[52]^ Also, an inactive behavior has been shown to impact neuromuscular control (18), and thus may affect the other adopted strategies they rely on. Hence, the differences noted between inactive NSCLBP and inactive healthy controls might be attributed to the above interpretation.

However, in terms to physical activity, the present study found that active individuals had better postural control compared to inactive subjects. Specifically, inactive individuals with NSCLBP demonstrated poorer static and dynamic postural control compared to their active peers. But for the healthy controls, inactive individuals had difficulty mainly in dynamic control during the PL direction as compared to active individuals. Comparable to our findings, adopting a sedentary lifestyle behavior has been identified as a risk factor for impaired postural control and increased risk of falls.^[53,54]^ Likewise, physical inactivity can impact functional performance in people without disabilities. Sedentary older adults have been shown to have poorer postural control than their more active peers.^[55]^ This decline in postural control, associated with physical inactivity, was thought to be a result of reduced muscle force/mass, decreased mobility, and disturbed somatosensory integration.^[56]^ Importantly, such decline can be reversed through increasing physical activity.^[55,56]^

In addition to the reported differences, this study demonstrated a moderate but significant dose-response relationship between physical activity level and postural control. The higher the physical activity, the better the postural control. In a recent review conducted on physical activity and functional limitations, a similar dose-response relationship was displayed such that those with higher levels of physical activity were less likely to develop functional limitations as compared to a sedentary group.^[57]^ The relationship between postural control and physical activity in NSCLBP population has been less studied. However, several studies have examined the relationship between physical activity and other outcome measures, including pain and disability and reported similar dose-response associations.^[14–16]^ A sedentary behavior was associated with increased physical disability, which can impact postural control.^[17]^

### 4.2 Hip muscle strength

The results indicated that hip muscle strength was significantly diminished in physically inactive individuals as compared to physically active peers. Hip muscle weakness has been associated with a wide range of lower extremity injuries and chronic diseases.^[58–60]^ In addition, weakness or inefficiency of hip muscles may lead to lumbopelvic imbalance, which can contribute to the development of LBP.^[61,62]^ Hip muscles, in particular the gluteus maximus, are tightly coupled to the lumbar paraspinal muscles (contralateral latissimus dorsi) via the thoracolumbar fascia, which facilitates the transfer of energy and load from the lumbar spine to the lower extremities.^[63,64]^ Thus, hip muscles have an important role in lumbar stability.^[65]^ Furthermore, hip muscles serve to maintain pelvic stability and control the rotational movement of the lower limbs during single leg stance.^[66]^ Hence, weakness in these muscles may cause decreased pelvic stability, leading to abnormal segmental movement of the lumbar spine during gait or standing, which may also contribute to LBP.^[67]^ However, the contribution of hip muscles weakness to LBP development is still controversial. While some studies have reported that hip muscles strength is diminished in LBP patients^[60,68]^, others have found no relationship between hip strength and the development of LBP.^[69,70]^ The current study found no significant differences in hip strength between individuals with NSCLBP and healthy controls. It should be noted, however, that NSCLBP is a complex and multifactorial process and thus could explain the difficulty in establishing specific differences. It is possible that diminished hip muscle strength is present in just a subgroup rather than in all LBP patients, meaning that some patients should not be expected to experience any change in muscle strength.

Irrespective, a difference in muscle strength by physical activity is still evident. Furthermore, a significant dose-response relationship between physical activity level and peak force of hip muscles was found. The lower the physical activity, the lower the strength. The decrease in muscle strength could be a factor for the impaired postural control seen in inactive subjects. Muscle weakness is a well-established risk factor for impaired postural control and increased risk of falls.^[71–75]^ Evidence suggests that in response to physical inactivity, skeletal muscles go through a process called adaptive reductive remodeling.^[76]^ This causes a loss of muscle mass (disuse atrophy), as a result of reduction in muscle fibers and loss of motor units,^[76]^ leading to decreased muscle strength. The reduced muscle strength may contribute to the diminished ability to meet the biomechanical requirements for postural control, which could have significant consequences on maintaining functional independence and ability to execute daily tasks. The YBT requires neuromuscular control through proper joint positioning as well as strength in the surrounding musculature to create and maintain the necessary positions throughout the test. Previous studies have shown a correlation between hip extensor strength and all three directions of the YBT.^[72,77,78]^ Reaching in these directions is usually accompanied by an anterior shift of the trunk to maintain the center of mass within the base of support. Flexion of the trunk produces flexion moment at the hip, which is controlled by the hip extensors.^[79]^ Reaching far may require further shift of the trunk anteriorly and stronger hip extensors to counteract this motion while maintaining stability. Inactive individuals in our study had weaker hip extensor strength and thus their ability to reach far might be limited due to the inability of the hip extensors to counteract the sagittal plane flexion of the trunk and hip. Thus, any attempt to reach far might cause them to lose control while performing the task.

In addition, Hubbard et al^[78]^ showed that the PM and PL reach distances on the Star Excursion Balance Test were correlated with hip abductor strength. Hip abductor strength works to stabilize the pelvis during the single leg stance activities by resisting the force of gravity on the unsupported reaching leg.^[66,71]^ Performance in the posterior directions requires lateral stabilization of the pelvis and thus differences in hip abductor strength may partly account for the variances in YBT reach distances noticed between active and inactive subjects. Inactive individuals in our study demonstrated weaker hip abductor strength as compared to their active peers. This weakness in hip abductor strength may cause a reduction in reach excursion as reaching far might require a greater lateral stability. Nonetheless, we did not find significant differences in external rotators strength between active and inactive individuals, which could be due to the smaller sample size to find such differences. However, in a study by Wilson et al,^[71]^ hip external rotation weakness was found to be less likely related to poor performance on the YBT.

Postural control is a complex autonomic phenomenon that occurs as result of integration of several body systems (visual, vestibular, and somatosensory or proprioceptive sensations).^[49]^ It relies on the integration of sensory (afferent) input with a motor (efferent) output that accurately matches the postural requirement to execute smooth and coordinated neuromuscular actions.^[49]^ With a sedentary behavior, these unconscious processes may not integrate as well or as quick as they would do when the person is active, and as a result, there might be an associated increase in static postural sway and a diminished dynamic stability. This can increase the risk of falls and injuries, limits functional performance, negatively or psychologically affect the person, and may worsen LBP symptoms. Therefore, maintaining an adequate physical activity level is crucial. Physical activity has been well documented and recognized in the international guidelines as one of the primary care strategies and a key element of the self-management of acute and CLBP.^[80–83]^ Physical activity, however, is a complex behavior and increasing physical activity is part of a behavior change process that focuses on targeting individuals’ social and physical environments.^[84]^ Being physically active is challenging and requires continuous motivation, a conductive environment, and consistent planning and scheduling.^[84]^ Despite the complexity of incorporating regular activity into a daily routine, it is a fundamental element in the rehabilitation of individuals with CLBP.

### 4.3 Limitations

Certain limitations must be taken into consideration when interpreting the results of this study. First, the use of self-reported questionnaire to assess subjects’ physical activity levels may allow patients to minimize or exaggerate the amount of physical activity performed. Participants were asked to state their physical activity level in the preceding week, which may also be questioned because of the risk of recall bias. Therefore, some participants may have been misclassified. However, to minimize this potential limitation, we excluded subjects who scored between 550 and 649 MET as well as assured that we had 2 heterogeneous groups in regards to physical activity levels to eliminate any potential biases on results.^[35]^ In contrast to questionnaires, assessment with activity monitors/devices (eg, wearable fitness tracker) may provide more accurate/reliable information. However, the IPAQ-SF was shown to be affordable, more feasible, and easy to apply in clinical settings. Second, timing of the assessments (morning vs afternoon) to examine postural control and strength was not controlled. Third, a median pain score of 3 for our cohort may be too low to produce significant differences. Also, other physical factors that may be associated with variations in performance such as neuromuscular control and range of motion of the joints (eg, closed kinetic chain ankle dorsiflexion range of motion) were not examined in this study. Thus, we cannot ascertain to what extent subjects’ limited reach capacity was associated with limited ankle dorsiflexion. Ideally, a subject would have 40 degrees of weight bearing dorsiflexion on each side with a minimum pass score of 35 degrees. Moreover, future studies should be performed using assessment methods of greater specificity, including the performance of the YBT over a force platform. Some measures of sway, such as the center of pressure parameters, maybe better at revealing specific differences than reach distance alone.^[35]^ In addition, because the design of this study was cross-sectional, the causality or directionality of the findings is uncertain. Also, a priori power analysis indicated that a sample size of 60 would be required to obtain a power of 80% using an effect size of (η^2^ = 0.10); however, a post hoc power analysis using the lowest obtained effect size when results were significant revealed that the power was 0.80 for postural control outcomes and 0.75 for strength. Lastly, because the sample size was small for a correlation analysis, larger prospective studies are required to establish the relationship between postural control and objectively measured physical activity in LBP population.

## 5 Conclusion

Based on our present findings, postural control was not significantly different between active individuals with and without NSCLBP. However, inactive individuals with NSCLBP exhibited diminished postural control compared to age-matched inactive healthy controls. Overall, physically inactive individuals had poorer postural control compared to their age matched physically active peers. Postural control and hip strength were independently related to physical activity behavior. In another words, a sedentary behavior may contribute to impaired muscle strength and postural control, and therefore impact functional performance in individuals with NSCLBP. The results of this study, however, should be interpreted with caution because of its cross-sectional design, and therefore causal relationships cannot be established. It is also important to consider other possible co-existing multi-dimensional factors that can affect postural control such as pain, kinesiophobia, and adoption of an alternate movement strategy.^[85]^ Thus, it can be assumed that there is no bivariate, but multivariate association, meaning physical activity is one of many factors affecting postural control.

### 5.1 Clinical Implications

Our findings suggest that positive outcomes could be gained by encouraging physically inactive persons to become more physically active. Therefore, more focus on the level of physical activity is needed both in formal healthcare settings and at home in daily activities. The results also suggest that improving hip strength could improve lumbopelvic stability and the performance on YBT and could potentially improve symptoms of LBP, decrease the risk of falls, and reduce future injuries. However, other muscles in the trunk, knee, and ankle may play an important role in frontal plane stability while performing the YBT and thus should be addressed. In summary, a physical activity program that focuses on increasing muscular strength, flexibility, postural control, and aerobic capacity would be beneficial for the rehabilitation of individuals with NSCLBP.^[86]^ However, caution should be taken with regards to dosage of physical activity, as too much activity can also be associated with LBP.^[86]^ and hence moderation is crucial.

## Acknowledgment

We would like to thank all participants who donated their valuable time to advance scientific inquiry.

## Author contributions

Muhsen B. Alsufiany contributed to the conception, participated in the design, managed the data collection, assisted in data interpretation, and contributed in writing and revising the manuscript. Everett B. Lohman contributed to the conception, participated in the design, provided administration, resources, and supervision, assisted in data interpretation and presentation, and substantially revised the manuscript. Noha S. Daher participated in the design, provided supervision, managed the data collection, performed the statistical analysis, assisted in data visualization, validation, and presentation, and substantially revised the manuscript. Gina R. Gang provided supervision and assisted in revising the manuscript. Amjad I. Shallan assisted with the data collection. Hatem M. Jaber contributed to the conception, participated in the design, provided supervision, managed the data collection, assisted in the statistical analysis, visualization, validation, interpretation, and presentation of the data, and was a major contributor in writing and revising the manuscript.
